# Biolayer Interferometry (BLI) to Quantify RALF1–Pectin Interactions

**DOI:** 10.21769/BioProtoc.5689

**Published:** 2026-05-20

**Authors:** Susan Lauw, Elke Barbez

**Affiliations:** 1 Core Facility Signalling Factory & Robotics, University of Freiburg, Freiburg im Breisgau, Germany; 2 Centre for Biological Signalling Studies (BIOSS), University of Freiburg, Freiburg im Breisgau,Germany; 3 Institute of Biology II, Molecular Plant Physiology (MoPP), Faculty of Biology, University of Freiburg, Freiburg, Germany; 4 Centre for Integrative Biological Signalling Studies, University of Freiburg, Freiburg, Germany

**Keywords:** Biolayer interferometry, Octet RED96, Peptide, RALF1, Oligogalacturonides, k_on_, k_off_, KD, R_max_, Sensorgrams, Savitzky–Golay Filtering

## Abstract

Cellular function relies on a network of precisely regulated interactions among macromolecules such as proteins, peptides, carbohydrates, and nucleic acids. These molecular interactions regulate vital processes, including signaling, structural organization, and developmental patterning. Biolayer interferometry (BLI) is a label-free optical biosensing technique that enables real-time quantification of such interactions. This protocol describes how to use BLI to assess the binding affinity between a biotinylated plant peptide hormone (RALF1) and cell wall–derived oligogalacturonides (OG25–50) on the Octet RED96 platform. Streptavidin-coated biosensors are employed to immobilize the ligand, while analyte binding is monitored through wavelength shifts in the reflected light. The protocol includes detailed steps for sensor preparation, assay setup, software configuration, and kinetic data analysis. While optimized for plant peptide–matrix interactions, the method is broadly adaptable to other macromolecular systems across biological disciplines.

Key features

• This protocol requires optimizing ligand and analyte concentrations to achieve optimal results.

• The buffer composition plays a vital role in supporting ligand–analyte binding, as their interaction is influenced by charge.

## Background

Biological systems depend on specific and dynamic interactions between macromolecules—including proteins, peptides, polysaccharides, and nucleic acids. These interactions are central to virtually all cellular functions, such as signal perception, transduction, structural organization, and developmental coordination. Whether mediating extracellular ligand recognition, intracellular signaling cascades, or mechanical integrity, the nature and strength of these macromolecular contacts shape cellular behavior. Accordingly, understanding how such interactions occur and how they are modulated is fundamental to dissecting biological function at the molecular level. In plants, the peptide hormone Rapid Alkalinization Factor 1 (RALF1) exemplifies how macromolecular interactions govern extracellular signaling. RALFs are peptide hormones that regulate diverse physiological processes, including organ growth, pollen tube guidance, and immune responses [1,2]. Our recent findings [3] demonstrate that RALF1 activity in roots critically depends on its ability to bind to de-methylesterified pectin, a negatively charged polysaccharide enriched in the plant cell wall. This charge-based association spatially localizes the positively charged peptide at the cell surface, enhancing downstream cellular signaling. In this context, de-methylesterified pectin acts not merely as a structural polymer, but as a dynamic signaling scaffold that modulates ligand availability and receptor activation, linking extracellular matrix state to intracellular signaling output. A variety of well-established techniques have long been used to study biomolecular interactions, including surface plasmon resonance (SPR), isothermal titration calorimetry (ITC), microscale thermophoresis (MST), and co-immunoprecipitation. These methods have greatly advanced our understanding of binding kinetics, affinities, and stoichiometries. While these approaches have proven highly valuable, they may face limitations in throughput, sensitivity, and compatibility with certain classes of molecules, particularly when dealing with highly charged, heterogeneous, or viscous macromolecular complexes. These limitations highlight the need for complementary techniques that are robust to complex sample matrices and can provide real-time, label-free kinetic data with moderate sample requirements.

Biolayer interferometry (BLI) measures binding events by detecting changes in the interference pattern of white light reflected from two surfaces within the biosensor: an internal reference layer and an external layer where the ligand is immobilized. When analytes bind to the ligand, the optical thickness of this external layer increases, causing a shift in the wavelength of the reflected light (Δλ). Because this shift is directly proportional to the mass accumulated on the sensor surface, BLI enables real-time, label-free quantification of association and dissociation kinetics. This physical principle underlies the technique’s sensitivity and explains why BLI is well-suited for studying charged or heterogeneous biomolecular complexes. In our previous study [3], we used BLI to quantitatively assess the interaction between biotinylated RALF1 peptides and oligogalacturonides (OGs) derived from de-methylesterified pectin. These measurements revealed a charge-dependent, high-avidity interaction, providing mechanistic insight into how extracellular matrix components modulate peptide signaling activity at the cell surface.

Here, we provide a detailed, step-by-step protocol for using BLI to measure the interaction between RALF peptides and pectin oligosaccharides. This optimized procedure allows researchers to assess macromolecular interactions involving charged biomolecules and can be broadly applied to study extracellular peptide–matrix binding events across biological systems.

**Figure 1.**
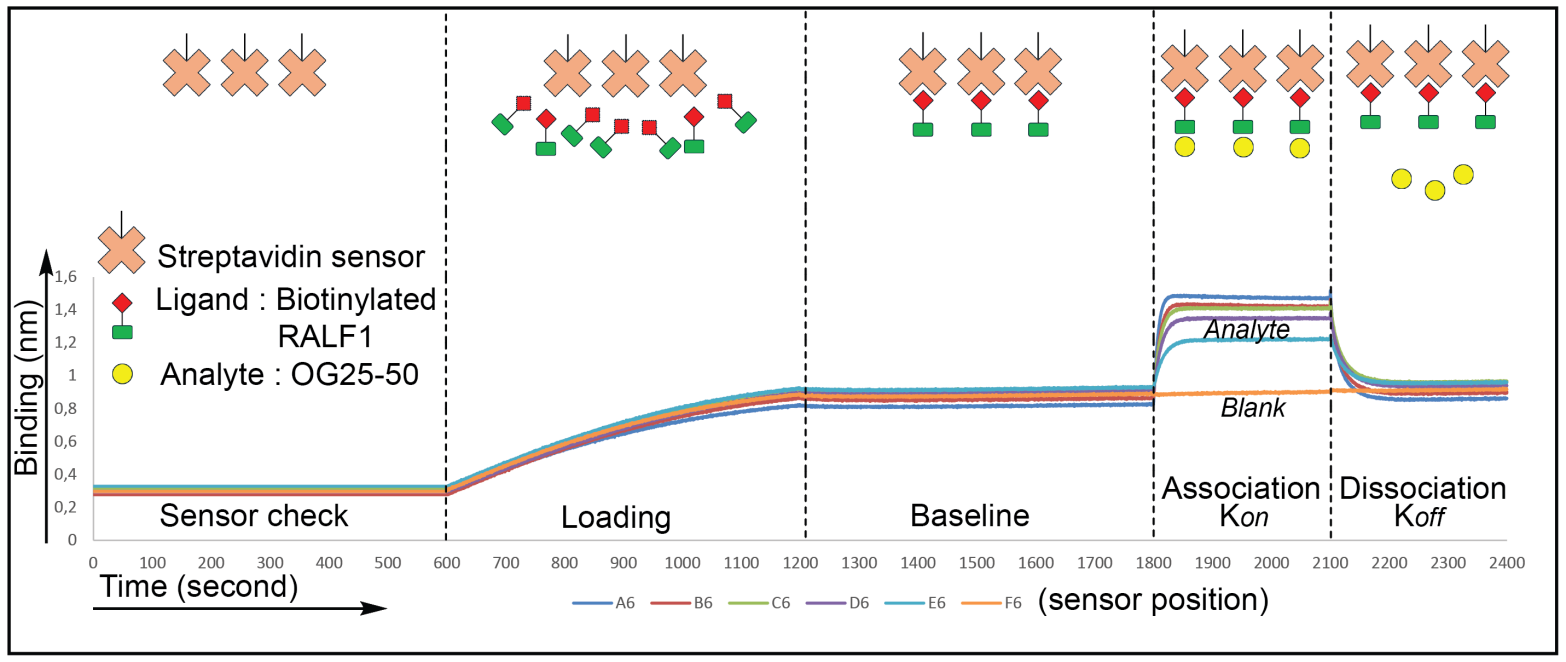
Schematic illustration of biolayer interferometry (BLI) setup to assess the binding between RALF1 and OG25–50

In this protocol, we use a streptavidin-coated biosensor tip that can be bound by a biotinylated ligand—in this case, a synthetic RALF1 peptide N-terminally conjugated with biotin. Once loaded with the ligand and washed to remove nonspecific binding, the sensor is sequentially dipped into wells containing buffer (for baseline), the analyte (pectin-derived OGs), and buffer again (for dissociation) (Figure 1). Binding of OGs to the immobilized RALF1 alters the interference pattern, and the instrument records the kinetics of association and dissociation in real time. These data allow quantification of the interaction’s affinity as well as association and dissociation rates, offering mechanistic insight into peptide–matrix interactions.

In BLI, a typical binding curve visualizes the change in optical thickness at the sensor tip as binding occurs between two molecules—most commonly a ligand immobilized on the sensor and a soluble analyte in solution. The curve consists of two main phases: the association phase, where the analyte binds to the ligand and the signal increases, and the dissociation phase, where the sensor is moved to analyte-free buffer and the signal declines as the complex dissociates. The shape and slope of the curve reflect binding kinetics (association and dissociation rates), while the amplitude correlates with binding affinity and analyte concentration. These kinetic traces form the basis for calculating key interaction parameters.

**Figure 2.**
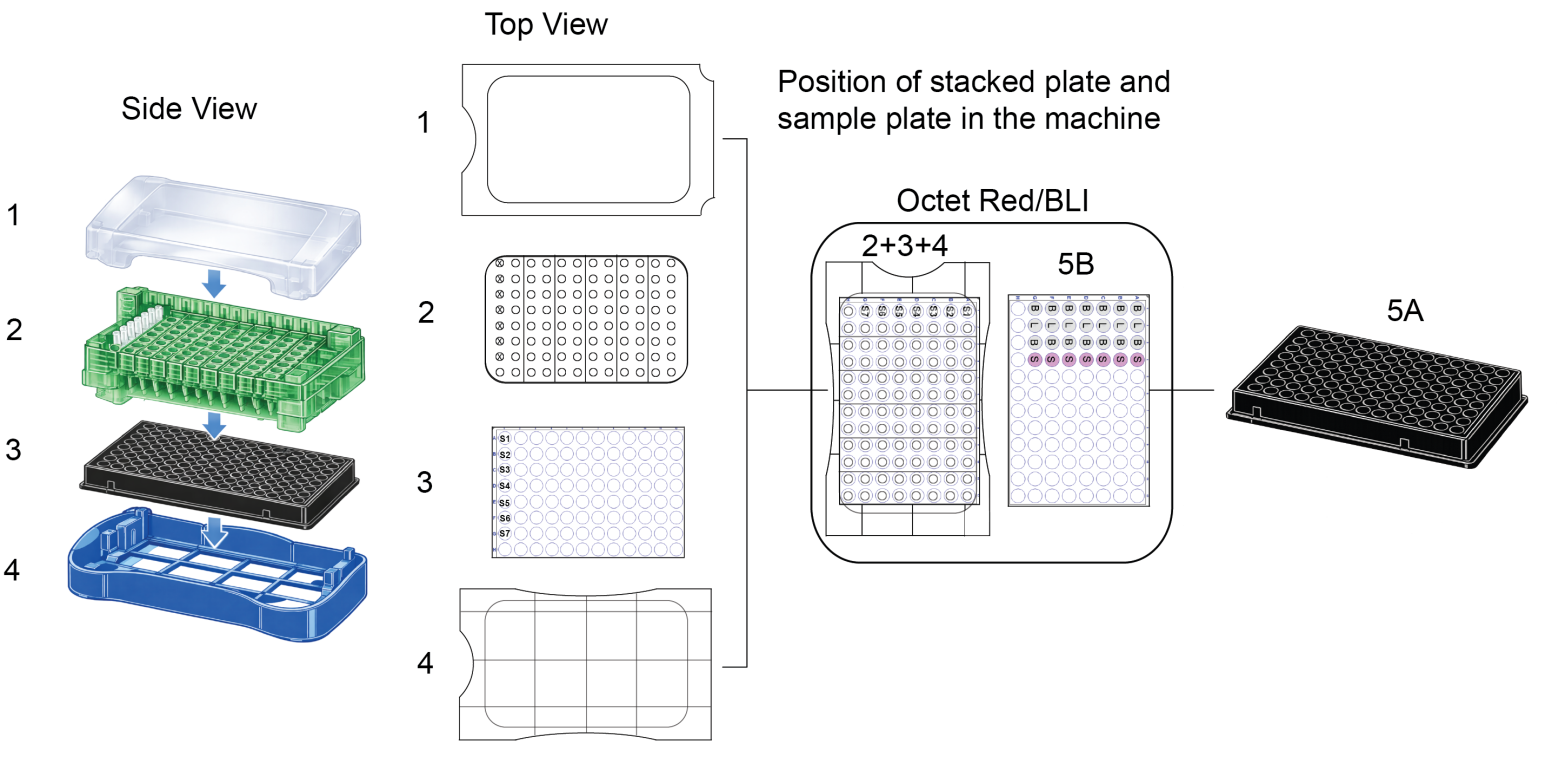
Assembly of the sensor rack and sample plate used for biolayer interferometry (BLI) measurements. (1) Lid. (2) Sensor rack with sensors. (3) Sensor plate. (4) Rack base. (5A) Overview of the sample plate. (5B) Positions of buffer and samples within the sample plate. The arrow indicates the order of assembly of the sensor rack components. The assembled sensor rack consists of the rack base, sensor plate, sensor rack, and sensors (2+3+4), which, together with the sample plate (5), are placed into the Octet BLI device. 3D representations are AI-generated (ChatGPT4.5) and subsequently edited in Adobe Illustrator.

We have structured the procedure into three distinct parts: (A) Sensor and sample preparation, which outlines all wet-lab steps needed before measurement; (B) software setup and running experiment, which guides users through programming the Octet instrument; and (C) data analysis. This separation allows users to prepare reagents and biosensors first, then configure the software interface, and finally launch the measurement, mirroring the actual experimental workflow in the lab. The schematic detail is shown in Figure 2, and the detailed protocol is described in the Procedure section.

In this assay, we use BLI to demonstrate that RALF1 binds OG25–50 with nanomolar affinity, while a charge-neutralized variant (RALF1-KR) does not bind, validating the assay’s sensitivity to electrostatic features of macromolecular interactions.

## Materials and reagents

**Reagents**

1. Biotinylated RALF1 peptide: synthetic, mature RALF1 peptide containing an N-terminal biotin modification; sequence: ATTKYISYQSLKRNSVPCSRRGASYYNCQNGAQANPYSRGCSKIARCRS; custom-synthesized (Peptide Specialty Laboratory GmbH, Heidelberg, Germany)

2. Charge-altered biotinylated RALF1-KR peptide: synthetic RALF1 peptide with reduced net positive charge containing an N-terminal biotin modification; sequence:

ATTSYISYQSLTSNSVPCSDTGASYYNCQNGAQANPYSDGCSYIASCRS; custom-synthesized (Peptide Specialty Laboratory GmbH, Heidelberg, Germany)

3. Pectin/galacturonan oligosaccharides (OGs), average DP 25-50 (Biosynth AG, Staad, Switzerland, catalog number: OG59705)

4. Dimethyl sulfoxide (DMSO) (Sigma-Aldrich, catalog number: 276855)

5. MilliQ water

6. Di-Potassium hydrogen phosphate (Carl Roth, catalog number: 6875.1)

7. Sodium dihydrogen phosphate dihydrate (Carl Roth, catalog number: T879.3)

8. Sodium chloride (Carl Roth, catalog number: 9265.2)

9. Potassium chloride (Carl Roth, catalog number: 6781.1)

10. Albumin fraction V/bovine serum albumin (BSA) (Carl Roth, catalog number: 8076.2)

11. BLI binding buffer: phosphate buffer saline (10 mM phosphate buffer, 137 mM NaCl, 2.7 mM KCl), pH 7.4, and 25 μg/mL BSA

**Laboratory supplies**

1. Octet^®^ streptavidin (SA) biosensors (Sartorius, catalog number: 18-5019)

2. Microplate 96 well, PS, F-bottom, black (Greiner, catalog number: 655900)

3. Microtube 1.5 mL (Sarstedt AG & Co.KG, catalog number: 72.690.001)

4. Microtube 2.0 mL (Sarstedt AG & Co.KG, catalog number: 72.691)

## Equipment

1. Biolayer Interferometry (Fortebio-PALL Life Sciences, Sartorius, model: Octet^®^ RED96)

## Software and datasets

1. Octet Data Acquisition software (Sartorius, version 8.0.2.5)

2. Octet Data Analysis software (Sartorius, version 8.0.2.3)

3. GraphPad Prism^®^ (Dotmatics, version 6.07)

## Procedure

Information on the measurement protocol is described in the Fortebio manuals (Fortebio Inc., 2011, Octet System Data Analysis User Guide, see [4]). Additional details on the streptavidin biosensor can be found in the Streptavidin (SA) biosensors guide (Fortebio Inc. [5]).

**A. Sensor and sample preparation**

**Figure 3.**
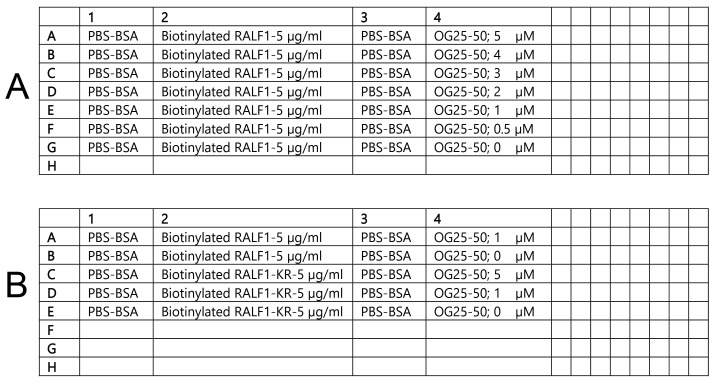
Solution positions in the sample plates. Two experimental setups are shown: (A) Sample plate layout for the binding analysis between RALF1 and OG25–50; (B) sample plate layout for the comparative binding analysis of RALF1 and RALF1-KR with OG25–50.

1. Prepare all components shown in Figure 2, including the plate lid (1), green sensor rack (2), sensor plate (3), rack base (4), and sample plate (5). Two plates are used for the BLI measurement: the sensor plate (3), which is used to soak the sensors prior to measurement, and the sample plate (5), which is used for sample analysis.

2. Place the required number of streptavidin biosensors into the green sensor rack (2). In the sensor plate (3), dispense 200 μL of BLI binding buffer into the wells corresponding to the sensor positions (see Figure 2). Keep the sensor rack (2) and sensor plate (3) separated while setting up the acquisition parameters in the software.

3. Prepare the ligand (of biotinylated RALF1/RALF1-KR) and analyte (OG25–50) solutions and fill the sample plate:

a. Dissolve the entire purchased vial of powdered RALF1 and RALF1-KR in DMSO to obtain 11 mg/mL stock solutions. Aliquot 20 μL portions into Eppendorf tubes and store at -20 °C prior to use. Dilute biotinylated RALF1 or RALF1-KR in BLI binding buffer to a working concentration of 5 μg/mL. Keep the solution on ice prior to pipetting onto the plate.

b. Dissolve the entire purchased vial of powdered OG25–50 in Milli-Q water to obtain 10 mg/mL stock solutions. Aliquot 50 μL portions into Eppendorf tubes and store at -20 °C prior to use. Prepare analyte solutions in BLI binding buffer at the following concentrations: 0, 0.5, 1, 2, 3, 4, and 5 μM. Keep the solutions on ice prior to pipetting onto the plate.

c. Begin assembling the 96-well flat-bottom black plate according to the layout shown in Figure 3, ensuring that all wells are prepared to a final volume of 200 μL.

d. In column 1, dispense 200 μL of BLI binding buffer (Figure 3). The BLI buffer in column 2 is used to establish the baseline for the sensors prior to loading.

e. In column 2, dispense 200 μL (5 μg/mL) of RALF1 or RALF1-KR into each well (Figure 3).

f. In column 3, dispense 200 μL of BLI binding buffer (Figure 3). BLI buffer in column 3 is used to measure the baseline after loading, as well as the dissociation step.

g. In column 4, dispense 200 μL of the OG25–50 solutions at their respective concentrations (Figure 3).

**B. Software setup and running experiment**

*Note: Switch on the Octet RED96 instrument ~40 min before the run to allow initialization and temperature equilibration. In the* Experiment Wizard, *select* Basic Kinetics *as the experiment mode.*

**Figure 4.**
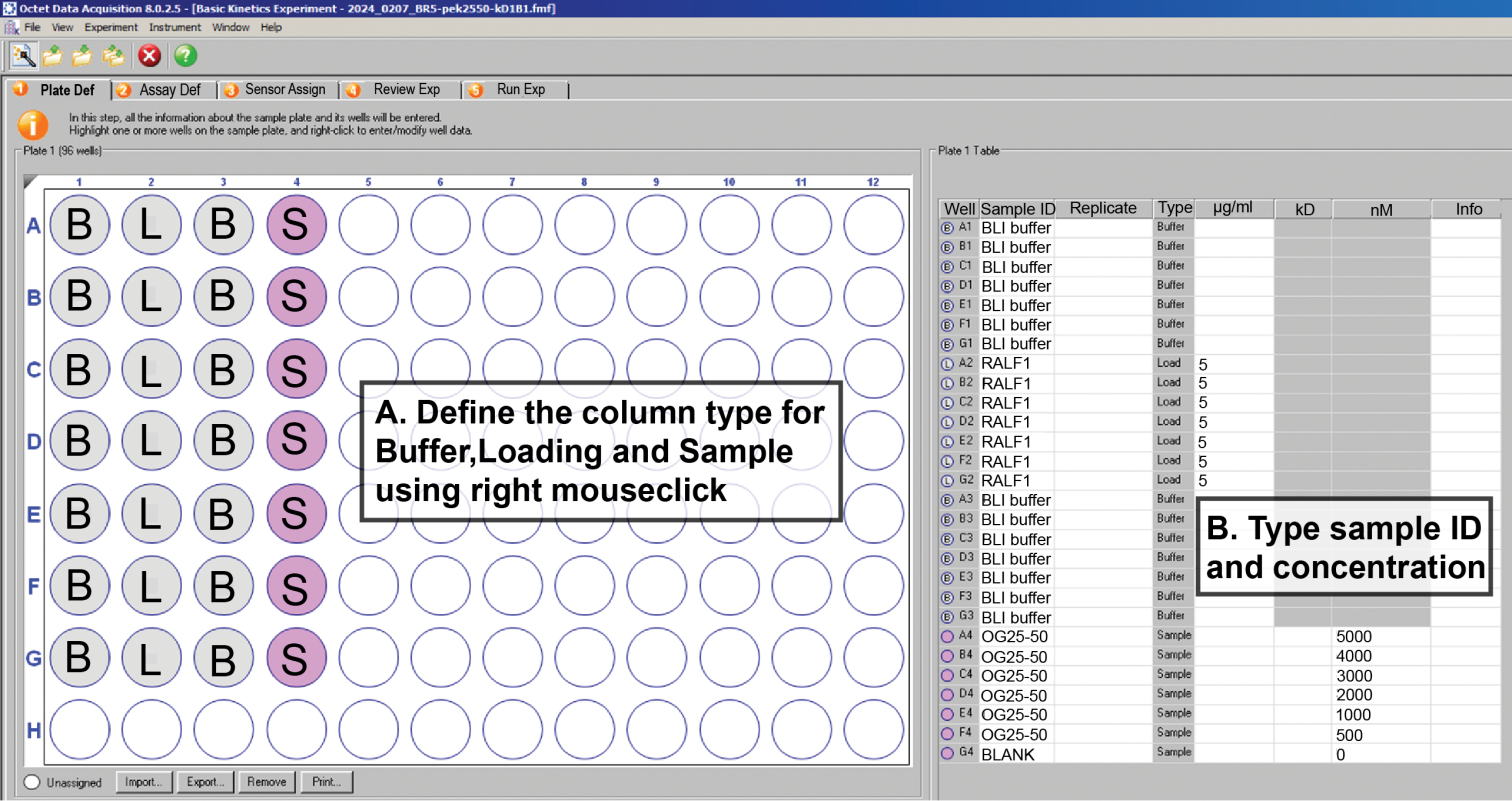
Plate definition setup in the Octet Data Acquisition software. Well types are assigned using the letters B (buffer), L (loading/ligand), and S (sample/analyte). The plate layout on the left shows the well assignments, while the table on the right lists the corresponding sample IDs, types, and concentrations entered for each position.

1. Plate definition

a. Open the *Plate Definition* tab and define the well types according to your sample layout (Figure 4). Use the right mouse button to assign each column as *Buffer, Loading*, or *Sample*, matching the positions of buffer, ligand, and analyte in the experimental plate.

b. Enter the Sample ID and the corresponding concentration for each well in the *Plate Table* on the right. Ensure that all entries accurately reflect the 96-well plate prepared in Section A so that the software setup matches the physical experiment.

**Figure 5.**
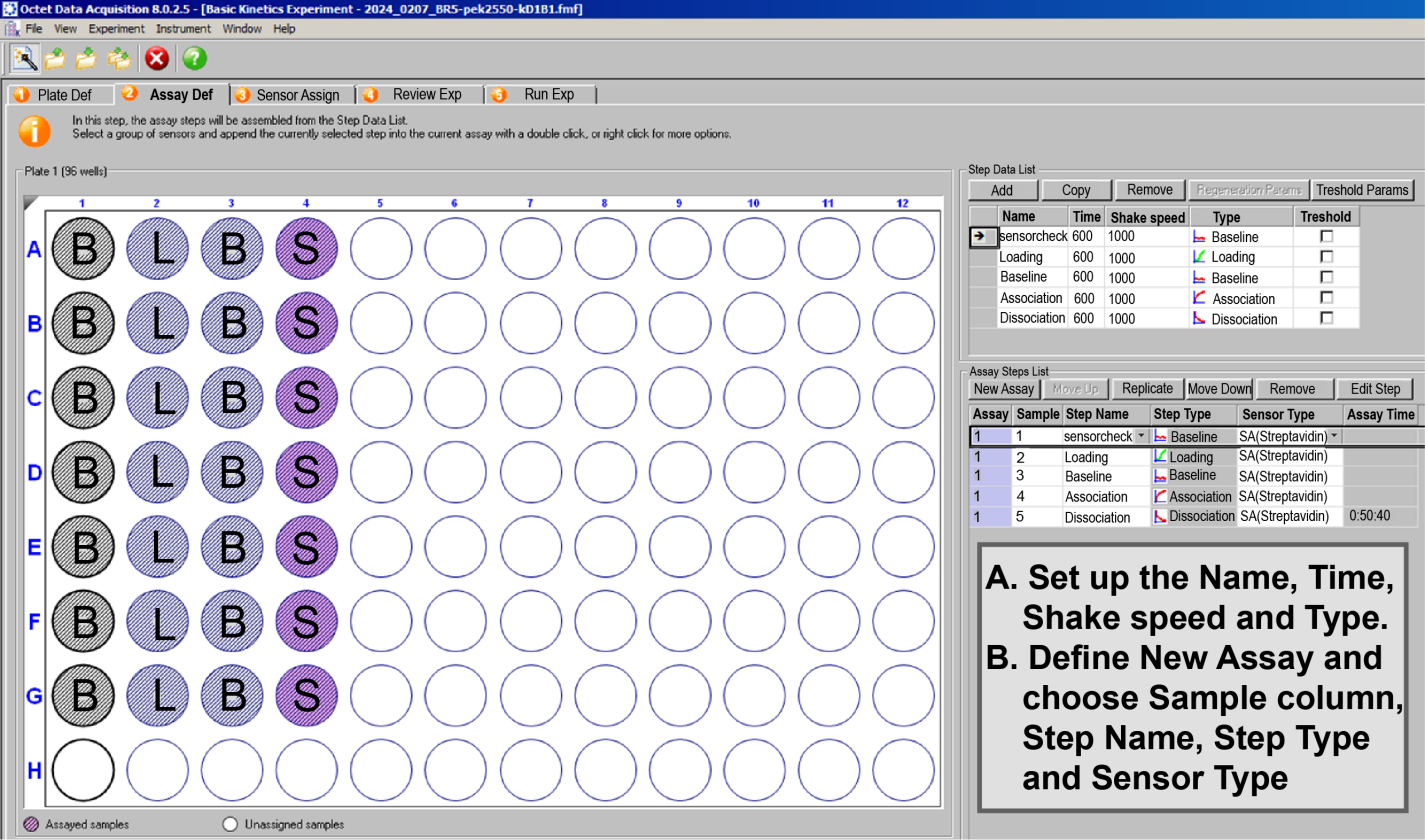
Assay definition. On the right, definition of measurement step and time.

2. Assay definition

a. In the Assay Definition tab (Figure 5), configure the kinetic assay by completing the Step Data List and the Assay Steps List.

b. In the Step Data List panel (upper right), define the individual steps that will be used to build the assay sequence. Create the following steps in the order shown in Figure 5: sensorcheck, Loading, Baseline, Association, and Dissociation, ensuring that the timing and shake speed parameters match the values displayed in the Step Data List. In this experiment, the Loading, Baseline, Association, and Dissociation steps are each set to a duration of 600 s, while the shake speed is kept at the default setting of 1000 rpm

c. In the Assay Steps List panel (lower right), assemble the predefined steps into the full experimental workflow. First, click New Assay to create a new sequence. Then, assign the appropriate Sample column, Step Name, Step Type, and Sensor Type for each step, as shown in Figure 5. Arrange the steps in the following order: sensorcheck → Loading → Baseline → Association → Dissociation. Finally, confirm that each step is linked to the correct well type (buffer, ligand, or analyte) as defined in the Plate Definition.

**Figure 6.**
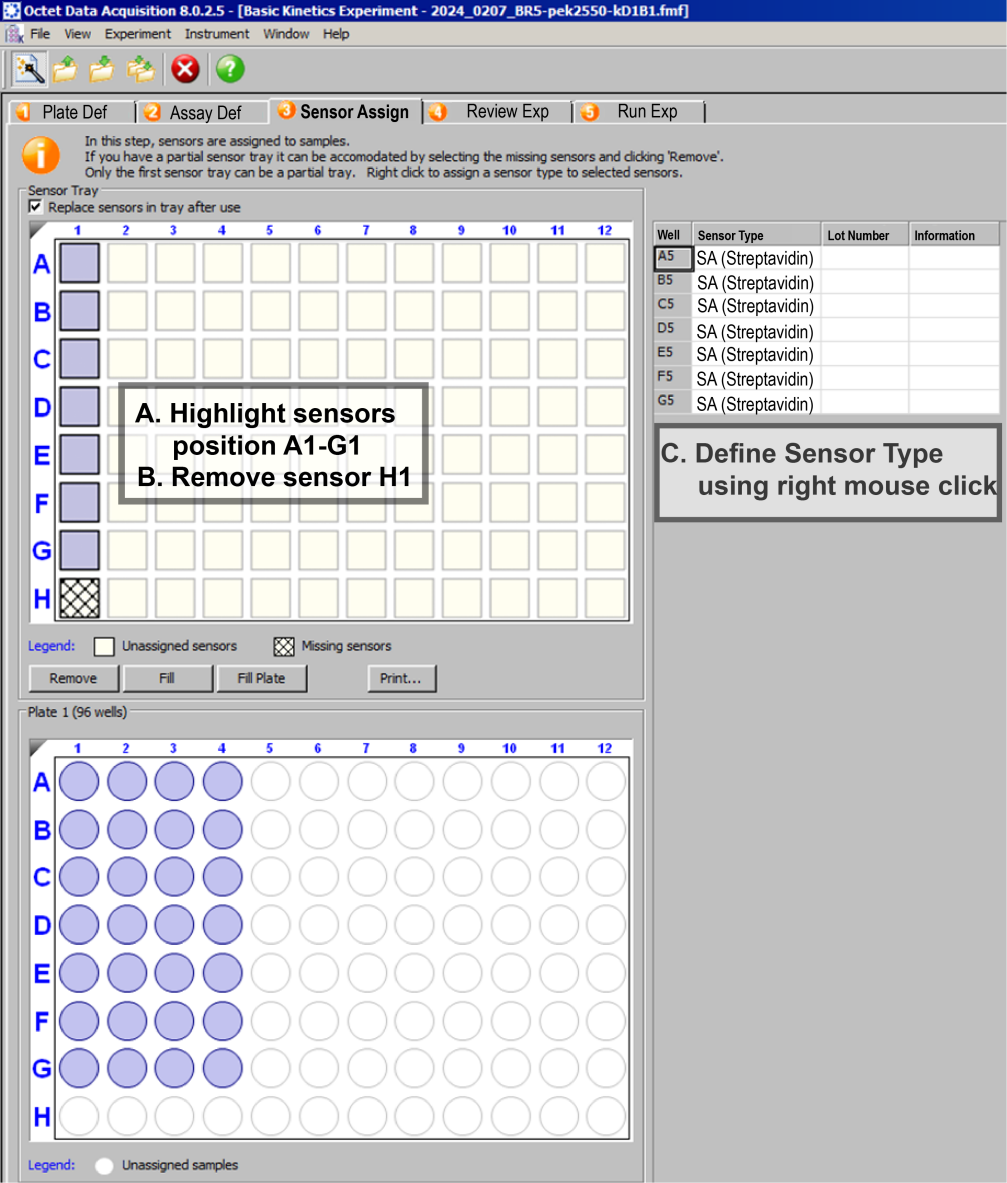
Sensor assignment in the Octet software

3. Sensor assignment. In the Sensor Assignment tab (Figure 6), assign the biosensors to the wells defined in the Plate Definition.

a. Select the positions for the sensors in the Sensor Tray. Highlight the sensors in positions A1–G1, as shown in Figure 6. Remove any unwanted or unused positions.

b. Right-click on the highlighted sensors to define the Sensor Type and select SA (streptavidin) from the list. The assigned sensors will appear in the Sensor Table on the right.

c. Ensure that the number and arrangement of sensors correspond exactly to the experimental layout prepared in Section A.

**Figure 7.**
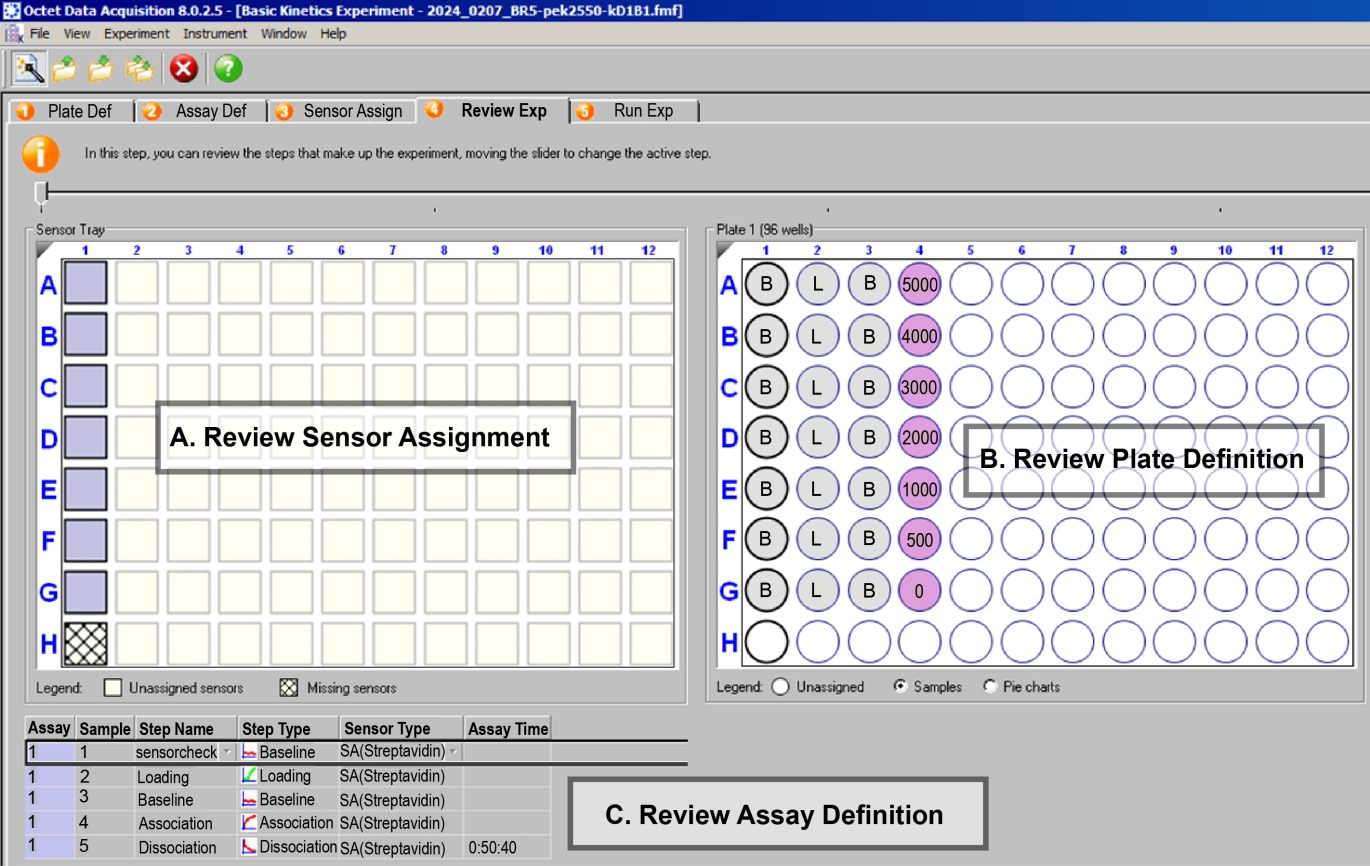
Review Experiment tab summarizes all configurations before the run

4. Review experiment. In the *Review Experiment* tab (Figure 7), verify that steps (A), (B), and (C) have been completed correctly.

a. Sensor Assignment (left panel): Confirm that sensors are assigned to positions A1–G1 and that unused sensors (e.g., H1) are removed.

b. Plate Definition (right panel): Check that buffer, ligand, and sample wells, as well as sample IDs and concentrations, match the layout prepared in Section A.

c. Assay Definition (bottom panel): Verify that the step order is correct: sensorcheck → Loading → Baseline → Association → Dissociation.

**Figure 8.**
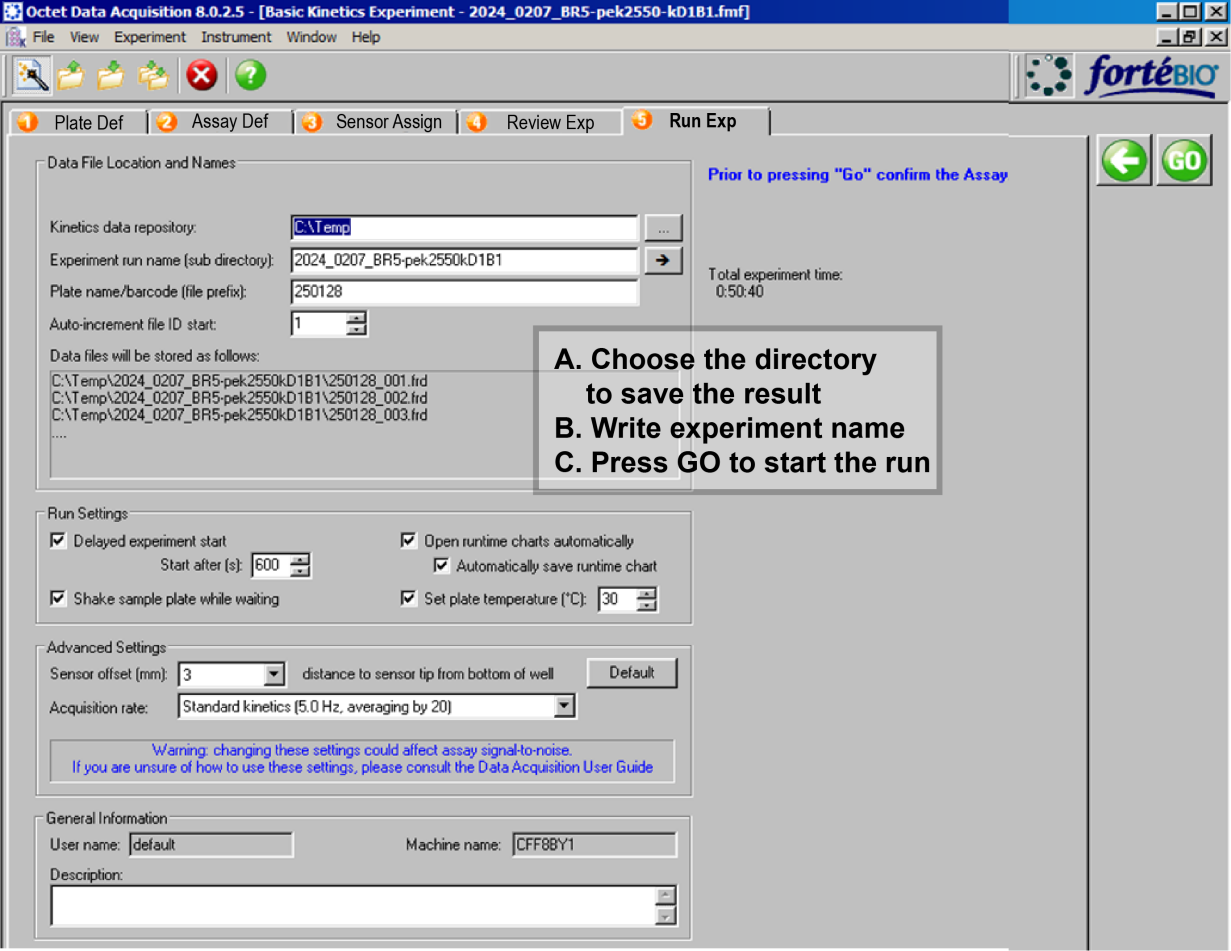
Run experiment. Location of data storage and name of file.

**Figure 9.**
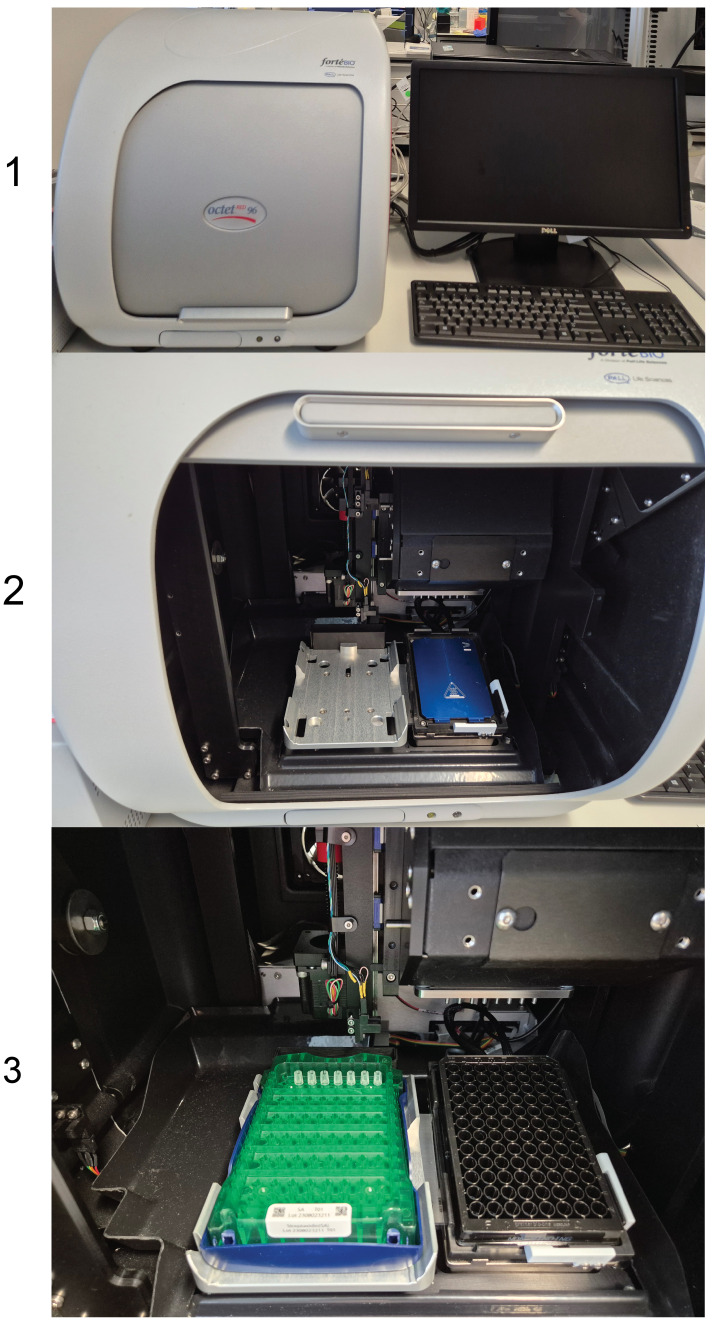
Loading the sensor stack and sample plate into the Octet RED96. (1) Octet RED96 instrument connected to a desktop computer. (2) Instrument in the open position. (3) Instrument with the stacked sensor rack and sample plate inserted, ready to start the run.

5. Running experiment

a. In the Run Experiment tab (Figure 8), choose the directory where the data will be saved and enter an experiment name and file prefix.

b. Review the run settings (plate temperature, shake mode, acquisition rate) to ensure they match your assay requirements.

c. Prepare the sensor hardware by stacking the sensor rack (including the pre-mounted sensors), sensor plate, and rack base as shown in Figure 2 (positions 2, 3, and 4).

d. Insert the stacked sensor hardware/left side and the prepared sample plate/right side (Figure 2, see position of stacked plate (2 + 3 + 4) and sample plate (5B) in the machine) into the matching position in the instrument as illustrated in Figure 9.

e. Press GO to start the measurement.

**C. Data analysis**

**Figure 10.**
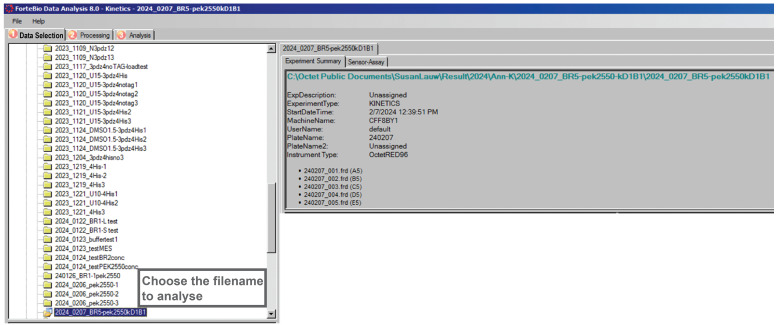
Data selection for analysis

1. Data selection

a. Launch the Octet Data Analysis software.

b. In the Data Selection window, navigate to the directory in which the experiment was saved and select the corresponding data file (Figure 10).

**Figure 11.**
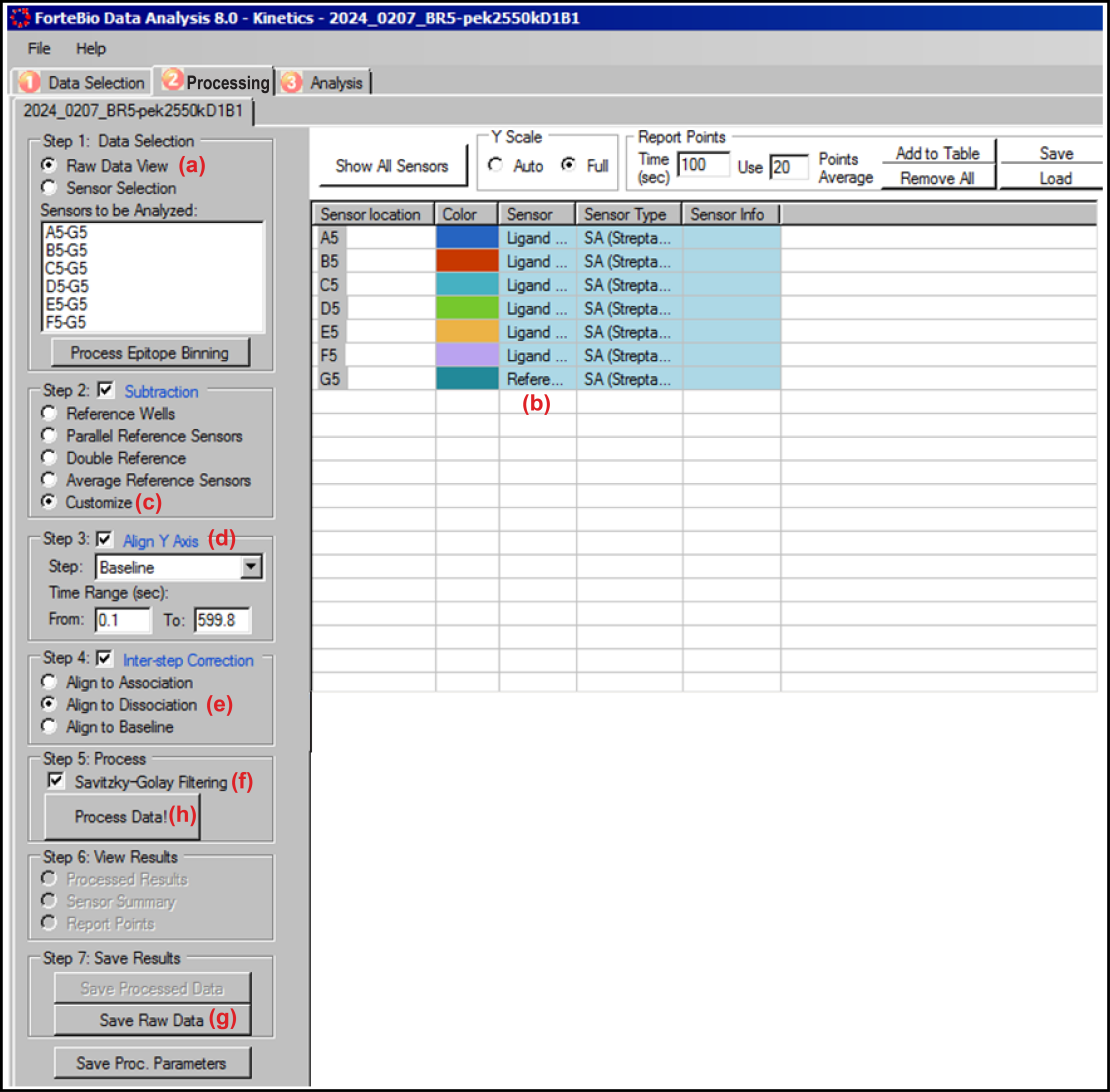
Data processing steps performed before kinetic analysis. (a) Data selection. (b) Sensor classification as ligand or reference sensors. (c) Performing subtraction. (d) Y-axis alignment step. (e) Inter-step correction. (f) Checkbox selection for Savitzky–Golay filtering. (g) Option to save raw data. (h) Execute data processing step.

**Figure 12.**
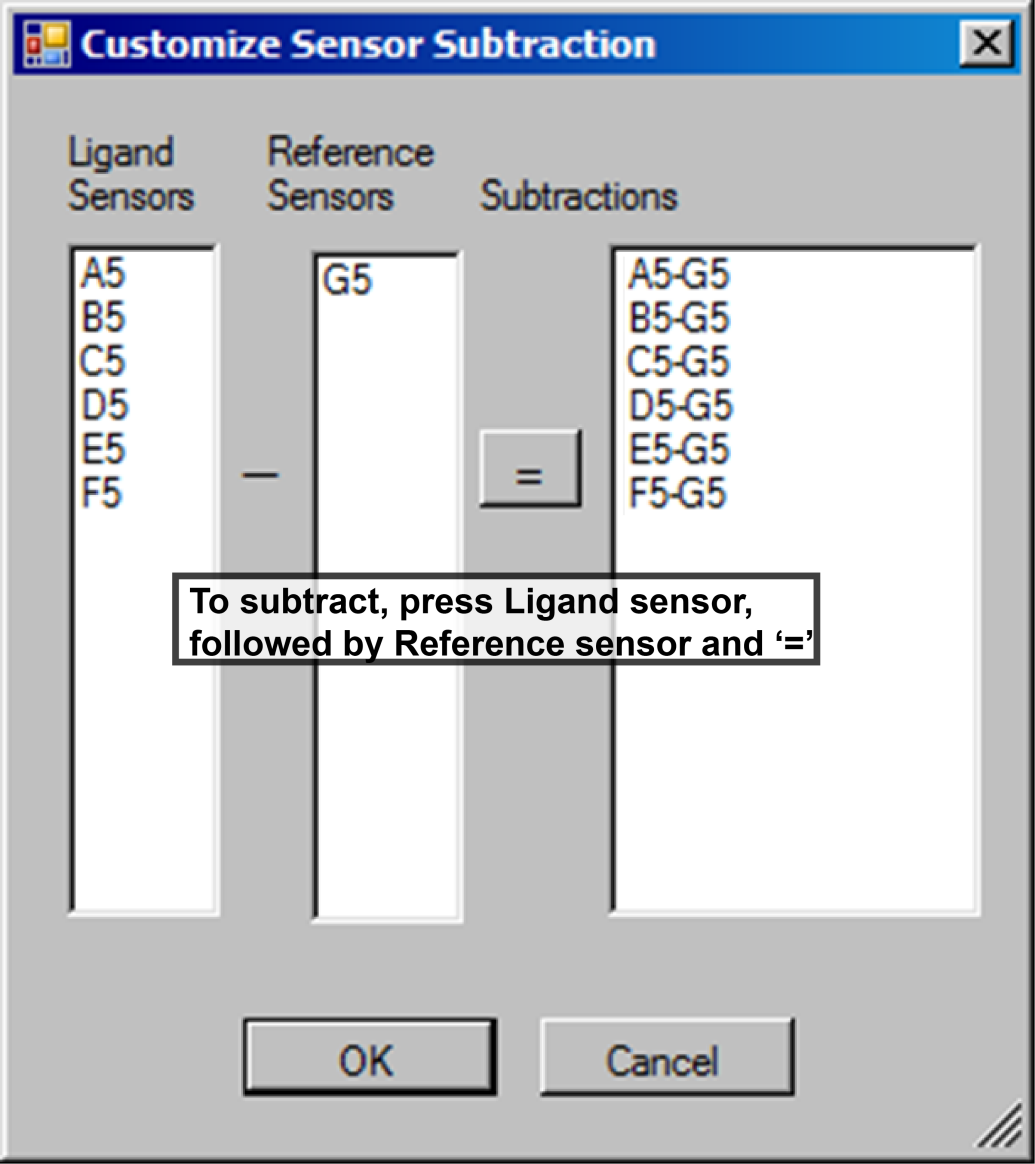
Sensor subtraction window

2. Data processing. Complete the steps below as shown in Figure 11:

a. Select Raw Data View to load the unprocessed sensorgrams (Figure 11a).

b. Assign each sensor as *Ligand* or *Reference* (Figure 11b).

c. Activate Customize (Figure 11c).

d. In the Customize Sensor Subtraction window (Figure 12), perform reference subtraction for each ligand sensor by selecting: Ligand sensor → ‘–’ → Reference sensor → ‘=’.

e. Each subtraction will appear in the Subtractions panel (Figure 12, right). This step removes nonspecific bulk signal and ensures that only ligand–analyte binding contributes to the response. Enable Align Y Axis (Figure 11d) to normalize all curves to a common zero baseline.

f. Select Align to Dissociation (Figure 11e) to correct for minor baseline offsets between kinetic steps.

g. Apply Savitzky–Golay Filtering (Figure 11f) to smooth noise while preserving the kinetic shape of the curves.

h. Inspect the processed traces in the View Results panel to verify correct subtraction and alignment.

i. Save the processed sensorgrams using Save Raw Data (Figure 11g) for recordkeeping and downstream fitting.

j. Click Process Data to transfer the corrected curves into the Analysis tab (Figure 11h).

**Figure 13.**
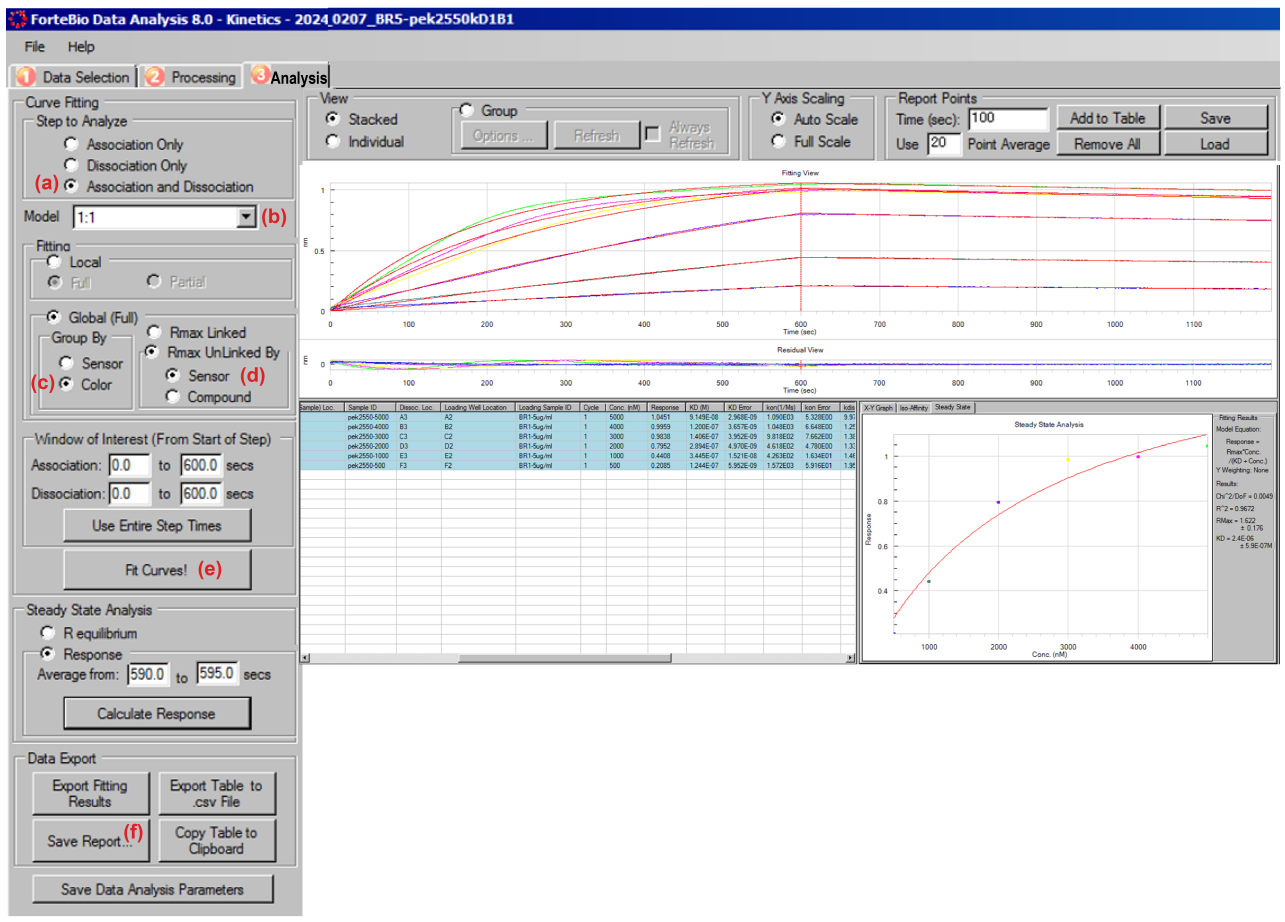
Analysis setup for kinetic curve fitting in the Octet Data Analysis software . (a) Selection of the step to be used for analysis. (b) Selection of the appropriate kinetic model. (c) Color-based selection of the global full group. (d) Rmax-linked sensor selection. (e) Choosing the fit curve. (f) Choosing save report.

3. Define analysis parameters. Complete the steps below as shown in Figure 13:

a. Select Association and Dissociation under Step to Analyze to include both phases of the sensorgram in the fit (Figure 13a).

b. Choose a 1:1 binding model from the Model dropdown menu to calculate k_on_, k_off_, and KD assuming a simple bimolecular interaction (Figure 13b).

c. Select Global (Full) fitting to fit all analyte concentrations simultaneously (Figure 13c). This approach increases robustness and is recommended for multi-concentration kinetic datasets.

d. Set Rmax Unlinked By Sensor to allow each sensor to have an independent maximum response value (Figure 13d).

e. Verify that the Association and Dissociation windows cover the full measurement period (default: 0–600 s for each).

f. Click Fit Curves! to generate kinetic fits, residuals, and extracted rate constants (Figure 13e).

g. Export the fitted results, including KD values, sensor-specific fits, residual plots, and fitting tables, using Save Report (Figure 13f).

**Figure 14.**
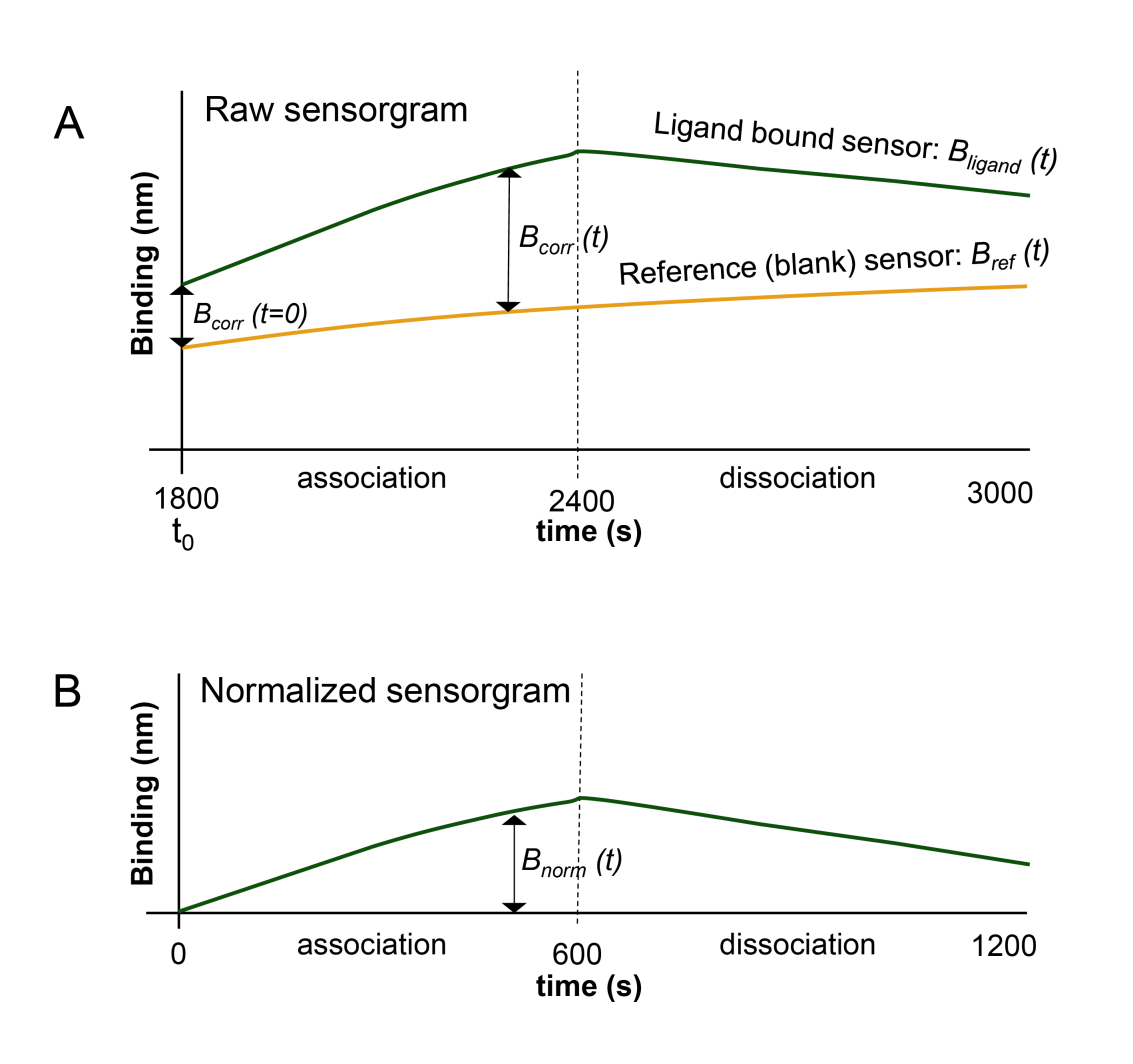
Schematic of data normalization and visualization steps. (A) Graph before normalization and associated steps. (B) Graph after normalization. The calculations for the normalization process are provided in the supplemental material.

4. Raw data normalization and GraphPad Prism visualization: To improve figure resolution and ensure proper annotation, the data are normalized and then transferred to GraphPad Prism for plotting. An overview of the steps is provided in Figure 14 and detailed below:

a. Reference subtraction: For each time point (t), the signal from the reference (blank) sensor is subtracted from the signal of the ligand-bound sensor.

*B_corr_ (t)= B_ligand _(t)-B_ref _(t)*

b. Baseline normalization: After reference subtraction, B_corr_ is expected to be 0 at time point t = 0. In practice, however, a small offset often remains, for example due to minor differences between sensors, imperfect reference matching, or slight baseline drift during measurement. To normalize the trace, the value of Bcorr(t = 0) is therefore subtracted from B_corr_ all time points (t).

*B_norm_(t)= B_corr _(t)-B_corr _(t_0_)*

c. Time normalization: Because the association and dissociation phases are the relevant parts of the BLI measurement, the time axis is shifted with the start of the association defined as time point 0. In other words, we reset the x-axis to 0.

*t_corr_=t-t_0_*

## Validation of protocol

**A. Binding analysis of RALF1 and OG25–50**

To confirm that the protocol reproduces known interaction behavior, we first applied it to the RALF1–OG25–50 system previously characterized in [3]. Under the conditions described here, RALF1 showed a strong and characteristic binding response to OG25–50, including a steep association phase and a stable plateau, consistent with earlier kinetic measurements.

**B. Binding analysis of RALF1-KR and OG25–50**

**Figure 15.**
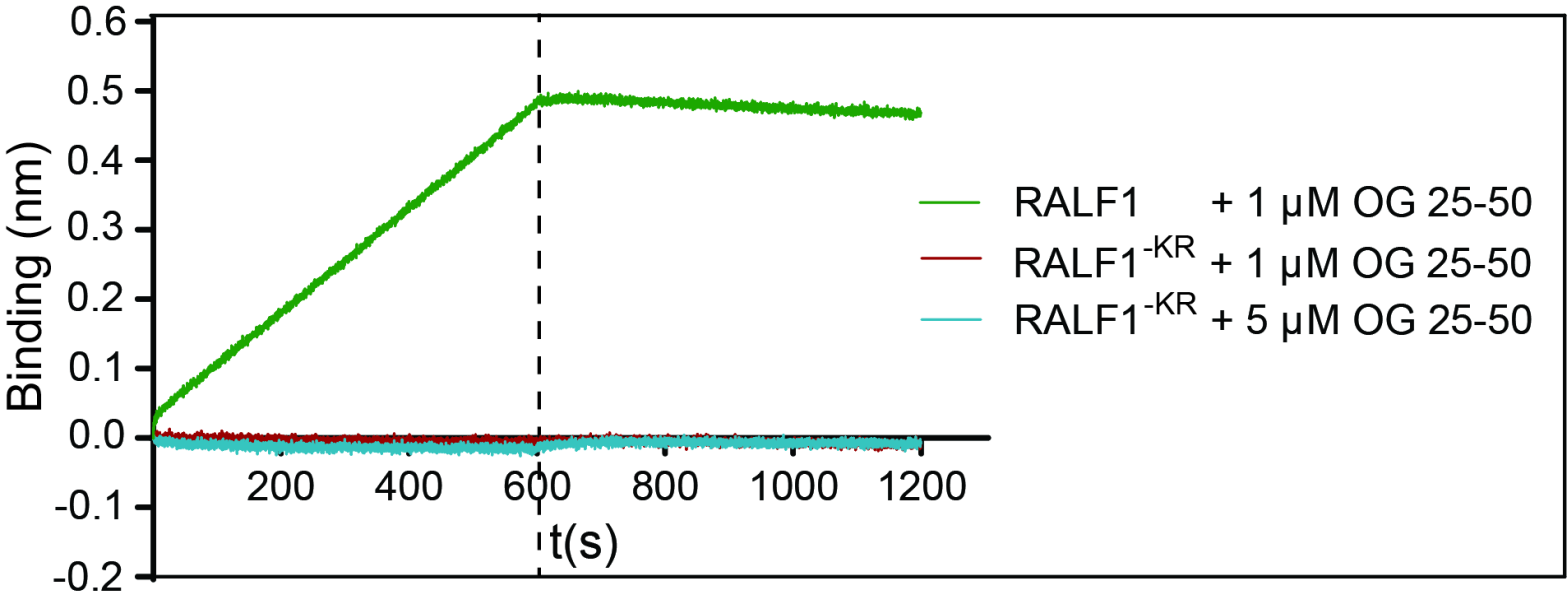
Biolayer interferometry (BLI) measurement between RALF1-KR/RALF1 (ligand) and OG25–50

We further validated the assay by reproducing a published comparison of RALF1 with a charge-neutralized mutant, RALF1-KR, as described in [3]. While wild-type RALF1 showed robust binding to OG25–50, RALF1-KR exhibited no detectable interaction at either 1 or 5 μM analyte, resulting in flat sensorgrams (Figure 15). This published dataset confirms that electrostatic properties are essential for RALF–pectin binding and demonstrates that the protocol sensitively distinguishes peptides with altered charge profiles.

## General notes and troubleshooting

**General notes**

1. The measurement requires optimization before performing the final measurements and analysis. Optimization parameters for BLI assay are as follows:

a. The Octet RED96 system uses only flat-bottom, 96-well black plates to reduce background interference and optical crosstalk.

b. Buffer composition (salt, BSA, pH): Buffer conditions strongly influence ligand–analyte binding, especially for charged molecules such as RALF peptides and OGs. Optimize pH, buffer strength, and salt concentration to identify conditions that support binding while minimizing background noise. BSA (25 μg/mL) is included to prevent nonspecific adsorption of peptides or OGs to the biosensor or plate surfaces. Prepare fresh buffer and use it within two days.

c. Concentration of ligand and analyte: Optimize ligand loading by testing concentrations between 0.5 and 20 μg/mL. Select a ligand concentration that yields sensorgrams approaching saturation during loading without exceeding recommended capture levels (0.5–2.0 nm). Use a range of analyte concentrations to obtain sensorgrams with well-defined association (the recommended range is 0.1–0.5 nm) and dissociation phases, enabling reliable global fitting and determination of kinetic parameters. A minimum signal-to-noise ratio (S/N) of 5:1 is recommended for appropriate analysis.

d. Timing for the baseline, association, and dissociation steps: Adjust the duration of each step so that the baseline is stable and flat, the association phase approaches saturation, and the dissociation phase extends long enough to observe clear decay kinetics. A well-optimized assay should yield similar KD values at different ligand loading levels and produce smooth, concentration-dependent association and dissociation curves suitable for global fitting.

e. Assess nonspecific binding using appropriate controls. The extent of nonspecific binding can be influenced by multiple factors. Examples and potential solutions include:

i. Ni-NTA sensors are prone to nonspecific binding of analytes lacking a His-tag. It is therefore recommended to include a control ligand with a comparable size and the same number of histidine residues in the His-tag.

ii. Streptavidin sensors have a lower propensity for nonspecific binding; however, it is still recommended to evaluate nonspecific interactions using a biotinylated negative control of similar size, in order to exclude nonspecific binding of the analyte to the streptavidin sensor during assay optimization.

iii. Analyte control: Use a molecule of similar size to assess nonspecific binding to the ligand.

iv. Electrostatic interactions are often nonspecific due to the general nature of charge–charge attraction. When a control ligand and an analyte have similar ionic properties, nonspecific binding is likely to occur. In the experiment comparing RALF1 and RALF1-KR binding to OG25–50, the results indicate that the positive charge of RALF1 is responsible for the observed interaction. Neutralization of this positive charge significantly reduced binding, resulting in a flat response. However, it cannot be excluded that positively charged RALF1 may also interact with other negatively charged compounds.

**Troubleshooting**

**Problem 1:** Post-regeneration measurements yielded inconsistent results.

Possible cause: Inefficient dissociation of the analyte from the immobilized ligand during repeated sensor reuse, leading to residual binding and altered sensor performance.

Recommended solution: Use streptavidin biosensors only once. Regeneration of SA sensors is not recommended—even during assay optimization—as partial analyte carryover can compromise baseline stability, reduce apparent binding capacity, and distort kinetic traces.
